# Mechanism of Enhancing Pyrazines in *Daqu* via Inoculating *Bacillus licheniformis* with Strains Specificity

**DOI:** 10.3390/foods12020304

**Published:** 2023-01-09

**Authors:** Qiuxiang Tang, Xiaoru Chen, Jun Huang, Suyi Zhang, Hui Qin, Yi Dong, Chao Wang, Xiaojun Wang, Chongde Wu, Yao Jin, Rongqing Zhou

**Affiliations:** 1 College of Biomass Science and Engineering, Sichuan University, Chengdu 610065, China; 2 Luzhou Laojiao Company Limited, Luzhou 646000, China; 3 National Engineering Research Centre of Solid-State Brewing, Luzhou 646000, China

**Keywords:** inoculation concentration, microbial community, metabolic pathway, pyrazines synthesis, strains specificity

## Abstract

Despite the importance of pyrazines in *Baijiu* flavor, inoculating functional strains to increase the contents of pyrazine in *Daqu* and how those interact with endogenic communities is not well characterized. The effects of inoculating *Bacillus licheniformis* with similar metabolic capacity on pyrazine and community structure were assessed in the *Daqu* complex system and compared with traditional *Daqu*. The fortification strategy increased the volatile metabolite content of *Daqu* by 52.40% and the pyrazine content by 655.99%. Meanwhile, results revealed that the pyrazine content in *Daqu* inoculated isolate J-49 was 2.35–7.41 times higher than isolate J-41. Both isolates have the almost same capability of 2,3-butanediol, a key precursor of pyrazine, in pure cultured systems. Since the membrane fatty acids of isolate J-49 contain unsaturated fatty acids, it enhances the response-ability to withstand complex environmental pressure, resulting in higher pyrazine content. PICRUSt2 suggested that the increase in pyrazine was related to the enzyme expression of nitrogen metabolism significantly increasing, which led to the enrichment of NH4^+^ and 2,3-butanediol (which increased by 615.89%). These results based on multi-dimensional approaches revealed the effect of functional bacteria enhancement on the attribution of *Daqu*, laid a methodological foundation regulating the microbial community structure and enhanced the target products by functional strains.

## 1. Introduction

*Daqu* (starter) is one of the essential raw materials and is also used to initiate the *Baijiu* fermentation. The yield and quality of fresh *Baijiu* are closely related to the composition of *Daqu* involved in various functional consortiums, enzymes, flavor constituents or their precursor [1,2,3]. In general, *Daqu* brewing *Baijiu* can be divided into three major types which are light, strong and sauce flavor [4]. Regulating the quality of *Daqu* is facing great challenges as it is controlled by artisans with their experiences and manufactured in an open environment, resulting in significant temporal and spatial characteristics [5,6]. *Muqu*, the high quality of the last year’s *Daqu*, was still added during the sauce-flavor *Daqu* manufacturing process [7]. With the help of genomics and bioinformatics, the dominant microbes and the correlation with the main metabolites were preliminary proved [8]. For example, *Thermoactinomycetaceae* and *Bacillaceae* dominated in high-temperature *Daqu* in which *Bacillus* was the core group [9]. *Bacillus* is also one of the dominant genera in both strong- and light-flavor *Daqu*; the former also includes *Aspergillus*, *Saccharomyces* and *Lactobacillus* [10], and the latter contains *Lactobacillus*, *Saccharomyces* and *Rhizopus* [11,12]. Mold affects the glucoamylase ability and liquefying ability of *Daqu*; meanwhile, it can avoid rancidity, and yeast is the main microorganism that affects fermentative capability and *Baijiu* production [13]. Bacteria mainly affect the flavor and quality of liquor [14].

These results laid an important foundation for developing new technology to improve the quality of *Daqu*. In recent years, the approaches to meliorate the *Daqu* focus on regulating process parameters or fortifying by functional strain/consortia. The former are mainly involved in regulating the change rate of bio-heating and humidity, which are the main driving forces to evolve the communities and their metabolism [15]. The latter can reconstruct the communities and their metabolization by inoculating the functional strain or consortia [16,17,18,19,20,21]. The contents of unique components, including tetramethylpyrazine, phenylethanol, etc., were remarkably enhanced by inoculating *Bacillus* spp. in *Daqu*, and it can also benefit the process of liquor fermentation [22,23]. Therefore, it was one of the hot spots on developing the technology of *Daqu* manufacture based on inoculating the functional strain or consortia in the last decades. *Bacillus licheniformis* was the dominant species isolated from high-temperature *Daqu*. The quality of high- and low-temperature *Daqu* were all improved when *B. licheniformis* was inoculated as the starter and endowed with their unique flavor [23,24]. The contents of aromatics, phenols, and pyrazines in fresh *Baijiu* were also increased by 2.4, 0.5, and 3.9 times, respectively, for the former, while the activity of amylase and the content of pyrazine and the aromatic compound was notably increased for the latter. However, there are few reports on the effect of inoculation intensity and strains specificity on the physicochemical property, community structure, and metabolites, even though these were unapplied in strong-flavor *Daqu* so far.

This study investigated the effect of the different initial concentrations of *B. licheniformis* in *Qupei* (to be cultured *Daqu*) on the physicochemical parameters, the community diversity, and their metabolome in strong-flavor *Daqu*. The research was dissected by polyphasic detection approaches, including conventional analyzing approaches, chromatography mass spectrometry, as well as the Illumina MiSeq platform, etc. The effects of the initial intensity on the network correlation among the communities and the relationship between functional microbes and main metabolites were inferred by bioinformatic technology. The aim is to reveal the effect of the inoculation intensity on the indigenous communities and their metabolism based on the isolate with strain specificity.

## 2. Materials and Methods

### 2.1. Strain Identification, Production and Collection of Daqu Samples

*B. licheniformis* J-41 and *B. licheniformis* J-49 were isolated from the *Muqu*, which was manufactured from *Daqu* powder undergoing a monthlong spaceflight in Shenzhou 11 spacecraft [25]. Genomic DNA from the strain was extracted using an Ezup Column Bacteria Genomic DNA Purification Kit (Shanghai Sangon Biotech, Shanghai, China). The detailed method described by Kakudo et al. [26] and its 16S rRNA gene was amplified by PCR with the forward primer 27F (5′-AGAGTTTGATCCTGGCTCAG-3′) and the reverse primer 1492R (5′-CTACGGCTACCTTGTTACGA-3′). Sequencing reactions were carried out using the dideoxy chain-termination method with an ABI 3730XL (Applied Biosystems) by Shanghai Sangon Biotech (Shanghai, China), and almost-complete 16S rRNA gene sequences were obtained. All sequencing data have been deposited at the National Center for Biotechnology Information. The GenBank accession numbers were SUB12491630 J-41 OQ135133 (*B. licheniformis* J-41) and SUB12491630 J-49 OQ135134 (*B. licheniformis* J-49). The cell membrane fatty acid extraction and analysis of two strains were based on the study by Zhang et al. [27].

In the process of making *Daqu*, firstly, the strain was inoculated into a commercial medium (LB, beef extract peptone medium), and the seed solution was prepared and incubated at 37 °C and 120 rpm/min for 24 h. Then, the suspension of 3 mL isolates was transferred to a 500 mL eggplant flask, which contained LB medium tilted by 100 mL agar and cultured at 37 °C for 24 h. Then, the suspension was prepared by eluting the culture medium with pre-prepared aseptic water. The number of suspension cells was counted by a blood cell meter and diluted to the corresponding concentration with tap water. *Qupei*, to be cultured *Daqu*, was inoculated with the suspension of both isolates, and the initial concentrations were 8 × 10^4^, 2 × 10^5^, 8 × 10^5^, 2 × 10^6^, 8 × 10^6^, and 2 × 10^7^ CFU/g (based on the dry material weight), which were numbered as A-1–A-6, and D-1–D-6, respectively. The blank sample without no inoculation was named B. These *Daqu* were produced according to the operation specification of *Daqu* manufacturing implemented by Luzhou Laojiao Co., Ltd. The *Daqu* (cultured for 30 days) was sampled according to the method described in the literature [28], and then, samples were sent to the laboratory for preservation (−20 °C and −80 °C) until further determination.

### 2.2. Determination Physiochemical Properties and Volatiles Constituents

The physiochemical properties, including moisture, acidity, liquefying ability, saccharifying ability, fermenting ability and esterifying ability, were determined according to the general methods of analysis for *Daqu* (QB/T 4257-2011) published by the Ministry of Industry and Information of the People’s Republic of China [29].

The extraction of flavor constituents in all samples was carried out by the headspace-solid phase microextraction (HS-SPME) method, using a 50/30 μm DVB/CAR/PDMS fiber (Supelco, Inc., Bellefonte, PA, USA). Firstly, took a 1.00 g sample into a 20 mL headspace bottle and simultaneously added 10 μL internal standard (0.0079 mg/100 mL, methyl octanoate). Then, samples were equilibrated at (60 ± 1) °C in a magnetic stirring plate for 15 min and extracted for 50 min. Finally, the fiber was immediately inserted the SPME fiber into the GC to thermally desorb the analytes at 250 °C for 5 min.

GC-MS (Gas Chromatography Coupled System TSQ 9000 Mass Spectrometer, Thermo trace 1300, Waltham, MA, USA) was equipped with an HPINNOWAX capillary column (30.0 m × 0.25 mm × 0.25 mm, Agilent Technologies Inc., Electron Corporation, Waltham, MA, USA). The detection protocol and data acquisition procedure were according to the method described previously with some modifications [30]. It was kept at 40 °C for 5 min, then increased to 100 °C at 4 °C/min for 0 min, and then increased to 230 °C for 6 °C/min and kept for 10 min. The inlet temperature was 270 °C, the ion source temperature was 300 °C and the mass spectrum scanning range was 35–400 m/z. Flavor components identification was compared with the mass spectrum data of those in the NIST2017 library database (Finnigan Co., San Jose, CA, USA) based on the following criterion: similarity (SI) > 800 (the highest value is 1000).

### 2.3. DNA Extraction, PCR Amplification, and Sequence Analysis of High-Throughput Sequencing

The total genomic DNA of *Daqu* was extracted using the Fast DNA SPIN extraction kit (MP Biomedicals, Santa Ana, CA, USA) following the manufacturer’s instructions; meanwhile, the DNA was quantified by a Nanodrop ND-1000 spectrophotometer (Thermo Fisher Scientific, Waltham, MA, USA) and stored at −20 °C prior to further analysis. The extraction quality was measured by 1.2% agarose gel electrophoresis, respectively. For bacteria, the V3–V4 domains of the 16S rRNA genes were amplified using primers 338F (5′-ACTCCTACGGGAGGCAGCA-3′) and 806R (5′-GGACTACHVGGGTWTCTAAT-3′). For fungi, the internal transcribed spacer ITS regions were amplified with primers ITS5 (5′-GGAAGTAAAAGTCGTAACAAGG-3′) and ITS1(5′-GCTGCGTTCTTCATCGATGC-3′). Sample-specific 7-bp barcodes were incorporated into the primers for multiplex sequencing. Specific PCR procedures were performed in accordance with the previous method [31]. The operating conditions of PCR amplification refer to the method of He et al. [28]. After the individual quantification step, amplicons were pooled in equal amounts, and pair-end 2 × 300 bp sequencing was performed using the Illumina MiSeq platform with a MiSeq Reagent Kit v3 at Shanghai Personal Biotechnology Co., Ltd. (Shanghai, China).

According to the Quantitative Insights Into Microbial Ecology (QIIME, version 2) pipeline, the low-quality sequences (length below 150 bp, average Phred scores less than 20, mononucleotide repeats over 8 bp, and ambiguous bases) were processed as previously described [32]. Finally, the DADA2 was used for quality control, denoise, splicing, and chimera detection to generate each deduplication sequence that was called amplicon sequence variants (ASVs) [33], and the abundance table of these sequences in each sample was referred to as the feature table, corresponding to the ASVs table.

### 2.4. Statistical Analysis

Significance analysis: using IBM SPSS Statistics 19 software (SPSS Inc. Chicago, IL, USA), Duncan’s test of one-way analysis of variance (ANOVA) was used to evaluate the significant differences between physicochemical and flavor compounds (*p* < 0.05, *n* = 3). Spearman correlation tests were performed for microbial genera and metabolites; *p* < 0.05 was considered a robust correlation.

Sequence data analyses were mainly performed using the QIIME2 and R software. The taxonomic tree [34] and principal coordinates analysis (PCoA) were carried out using QIIME2 and R software. The bacterial phylogenetic tree was constructed using QIIME2 [35,36]. The microbial functions were predicted using PICRUSt2 (Phylogenetic Investigation of Communities by Reconstruction of Unobserved States) [37] based on the KEGG PATHWAY Database [38,39,40]. The correlation analysis of physiochemical properties, metabolites, and ECs was analyzed by calculating Pearson’s rank. The correlations between microbes were analyzed by calculating Spearman’s rank with |RHO| > 0.5 and *p* < 0.01, which were visualized as a co-occurrence network using Cytoscape (version 3.7.2) [40]. A functional prediction using the functional annotation of prokaryotic taxa (FAPROTAX) and the database of metagenomics of bacterial community was used to identify ecosystem functions [41,42]. The FAPROTAX of bacterial communities was performed on the Tutools platform (https://www.cloudtutu.com (accessed on 13 September 2022)).

## 3. Results

### 3.1. Effect of the Initial Concentration in Qupei on the Physicochemical Properties of Daqu

As shown in Table 1, the physicochemical properties of *Daqu* were significantly changed by inoculation with *B. licheniformis*. The properties such as acidity, liquefying ability, saccharifying ability, and fermenting ability of *Daqu* were improved, but there was a non-linear correlation with the initial concentration. As the initial concentration increased, the esterifying ability was reduced, which in both D-2 and D-3 was less than half in control *Daqu* (B), while their fermenting ability was higher. It might be related to the ratio of liquefying ability to saccharifying ability (L/S). For example, the fermenting ability in both sample D-3 and sample D-4 was higher as the ratio of L/S was about 1.40, while the ratio of L/S in sample D-6 was 1.86, similar to sample B (1.82). It inferred that the balance of saccharifying and liquefying rate might be broken, resulting in the performance of mass and heat transfer varying due to the saccharification delayed. It may be one reason that the microbial community constituents and their metabolites shifted as the difference in the initial concentration changed.

### 3.2. Contents of Volatiles Increased as the Inoculation Intensity

**Figure 1:**
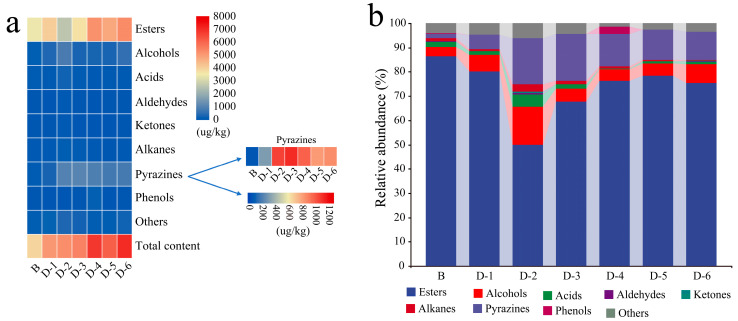
Volatile metabolites (**a**) content and (**b**) abundance of *Daqu*.

A total of 67 different volatiles were detected in these *Daqu*, including esters, alcohols, acids, aldehydes, pyrazines, ketones, phenols, alkanes, and other constituents, with a total content of 3603.66–7062.83 μg/kg (Figure 1). Recent studies reported that the content of volatile metabolites increased by *Bacillus* significantly in fortified *Daqu* [23,24]. Here, it was noteworthy that their amplitude was closely related to the initial concentration of *B. licheniformis* inoculated in the present experiment. For example, the content of volatiles was about two times higher than that of sample B when the initial concentration in *Qupei* ranged between 2 × 10^6^ and 2 × 10^7^ CFU/g. The amount of enhanced volatiles included in esters, alcohols, and pyrazines was 619.84–2229.62 μg/kg, 157.73–681.59 μg/kg, and 227.55–981.66 μg/kg, respectively. Among the volatiles detected, the proportion of esters was more than 50%, and methyl esters (methyl hexadecanoate, methyl caproate, methyl nonanoate, methyl dodecanoate, methyl stearate, etc.) were dominant constituents. The content of esters in sample D-6 was 1.72 times higher than that in sample B, while that of sample D-2 was slightly lower than that of sample B. From sample D-3 to sample D-6, the content of 2,3-butanediol and phenylethanol increased by 384.37–854.75% and 48.29–309.41%; these two kinds of constituents were endowed with creamy and rose aroma to *Baijiu* [43]. The content of pyrazines in sample D-3 was the highest; trimethylpyrazine and 2,5-dimethylpyrazine increased by 679.59% and 1664.27%, respectively. Additionally, 2,3-butanediol and phenylethanol also increased by 615.89% and 48.29%. Pyrazines not only can endow *Baijiu* with a charred aroma, but they are also beneficial to human health [44]. 3-Methylbutyric acid was only detected in sample D-2, sample D-3 and sample D-5, and the content was in the range of 26.75–103.99 μg/kg. 3-Methylbutyric acid is the precursor of ethyl 3-methylbutyrate, and the latter can endow the fruit and malt aroma of *Baijiu* [45]. At the same time, caryophyllene was detected in sample D-1, sample D-5 and sample D-6, and the content was 21.02–37.02 μg/kg.

### 3.3. Shifted Community Structure and Improved Pyrazine Content by Biofortification

Biofortification markedly affected the α-diversity index of *Daqu* (Table 2), but the amplitude was not linear as the inoculated initial concentration. By inoculating *B. licheniformis*, the community structure of *Daqu* was distinctly shifted, especially the bacterial community. The richness and diversity of the bacterial community were decreased when the initial concentration ranged from 8 × 10^4^ CFU/g (D-1) to 2 × 10^6^ CFU/g (D-4), while it increased at 8 × 10^6^ CFU/g (D-5) and 2 × 10^7^ CFU/g (D-6). The richness and diversity of the fungal community were decreased, and they were all lower than that in sample B, except the richness in sample D-2 was increased.

**Figure 2:**
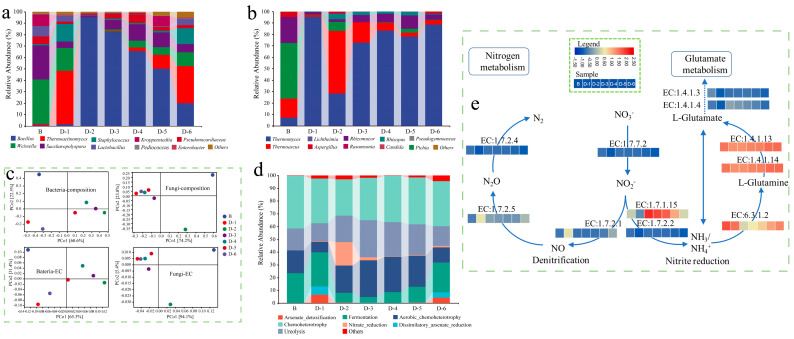
*Daqu* (**a**) bacterial and (**b**) fungal community composition, (**c**) principal coordinates analysis (PCoA) analysis of microbial and functional unit composition, (**d**) functional abundance of bacterial community based on FAPROTAX annotation, (**e**) nitrogen metabolism pathway in bacterial communities.

The composition profiles of microorganisms and functional modules in *Daqu* are shown in Figure 2. *Weissella*, *Saccharopolyspora* and *Kroppenstedtia* were dominated in sample B, and their abundances were 38.97%, 29.91%, and 10.17%, respectively, which was similar to the results reported by Yang et al. [46]. The initial concentration of *B. licheniformis* significantly affected the abundance of dominant microbes, and it especially increased the content of *Bacillus*. The ratio of *Bacillus* in FD ranged from 1.28% to 95.04%, but the amplitude was non-linear to the initial concentration, while that in sample B was only 0.66%. For example, the abundance of *Bacillus* in sample D-2 and sample D-3 occupied dominated absolutely even though the initial concentration of *B. licheniformis* inoculated was 2 × 10^5^ CFU/g and 8 × 10^5^ CFU/g. Conversely, the abundance of *Bacillus* in sample D-6 was about one-fifth in sample D-2, although the initial concentration of *B. licheniformis* inoculated in the former was 100 times higher than that in the latter. By inoculating *B. licheniformis*, *Thermomyces* and *Thermoascus* dominated in *Daqu*, and their abundances ranged from 81.52% to 96.97%, except for 28.21% in sample D-2, while *Pichia* and *Rhizomucor* dominated in sample B (Figure 2b).

PCoA analysis showed that the composition of the community and function in FD was significantly changed, which lay in the initial concentration of *B. licheniformis* (Figure 2c). The bacterial constructures and function in sample D-1 and sample D-6 were similar, while those in sample D-2 and sample D-3 were also similar. Analogously, the fungal communities and ecological function of FDs were shifted in contrast to sample B. The distance among FDs was closed except for sample D-2 as the abundance of *Thermomyces* was markedly lower than that in other FDs. The results suggested that the effect of initial concentration on the bacterial community and their function was more sensitive than the effect on the fungus’.

The community function database based on FAPROTAX annotation was used to analyze the bacterial community function in *Daqu* [41,42]. The results showed that the inoculation of *B. licheniformis* significantly changed the main bacterial functional composition (Figure 2d). Chemoheterotrophy, ureolysis, fermentation, and aerobic chemoheterotrophy dominated in sample B, while arsenate detoxification, dissimilatory arsenate reduction, and nitrate reduction had dominance in FDs. The ratio of these functional compositions in sample D-1 was similar to that in D-6. The abundance of nitrate reduction in both sample D-2 and sample D-3 was higher, as they are involved in nitrogen metabolism. It suggested that the expression abundance of EC:1.7.1.15 in the bacterial community in these FDs was enhanced based on the result predicted by the metabolic pathway of KEGG (Figure 2e). In contrast, the abundance of EC:1.4.1.13 and EC:1.4.1.4 in sample B was significantly higher than that in FDs, which contributed glutamate metabolism consuming NH4^+^. It was inferred that the effect of *B. licheniformis* was an accumulation of NH4^+^, which was beneficial to the biosynthesis of pyrazine.

**Figure 3:**
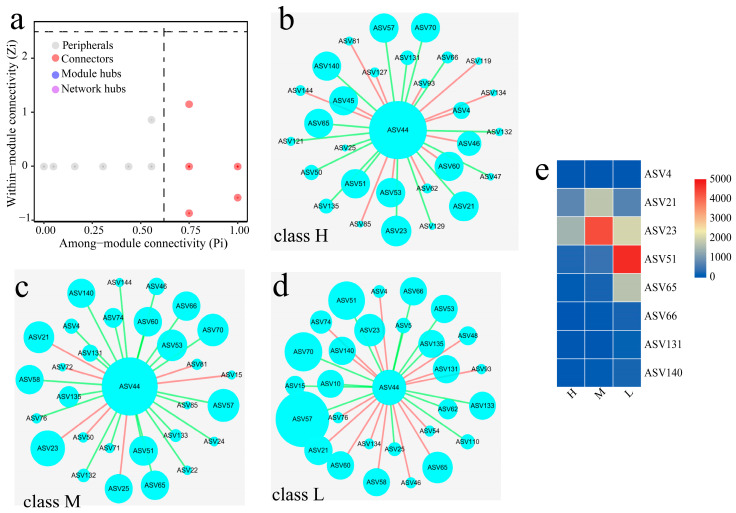
Analysis of *Daqu* bacterial network characteristics. (**a**) Zi-Pi of bacteria. (**b**–**d**) represented the co-occurrence network of class H, class M, and class L, respectively. (**e**) Heat map of microbe content. ASV4: *Bifidobacterium*, ASV21: *Pseudonocardiaceae*, ASV23: *Saccharopolyspora*, ASV44: *Bacillus*, ASV51: *Staphylococcus*, ASV65: *Lactobacillus*, ASV66: *Pediococcus*, ASV131: *Enterobacter*, ASV140: unclassified_Enterobacteriaceae.

The contribution of the inoculation intensity to the bacterial community and their function can be divided into three types based on the influencing pattern. They were H (D-2 and D-3), M (D-4 and D-5) and L (D-1 and D-6), respectively, and their network characteristics are shown in Figure 3. The most genera of bacteria were located in the peripheral region (Zi < 2.5, Pi < 0.62); only some of them belonged to Connectors (Zi < 2.5, Pi > 0.62) and have the function to connecting different microbial modules in the community. The results of the top 30 genera in the community demonstrated that 16 genera were shared among the three groups, and many genera had the function of Connectors. The results of heat-map analysis showed that among these microbes defined as Connectors, the abundance of ASV21 (*Pseudonocardiaceae*), ASV23 (*Saccharopolyspora*), ASV51 (*Staphylococcus*) and ASV65 (*Lactobacillus*) varied markedly in different types. The relationship between ASV44 (*Bacillus*) and ASV21 was similar to that of ASV44 and ASV23, and it was negative in the class H and positive in the class M and L.

### 3.4. Correlation between Fermentation Parameters, Metabolites and Community Function

**Figure 4:**
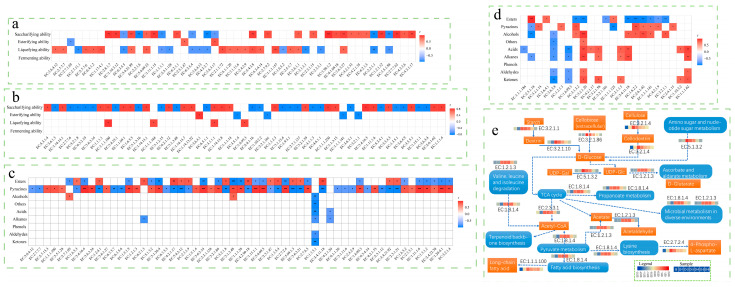
Correlation between fermentation parameters and enzymes: (**a**) Bacteria, (**b**) Fungi. Correlation between volatiles and enzymes: (**c**) Bacteria, (**d**) Fungi. (**e**) Main metabolic pathways in *Daqu* bacterial communities. Significance level labeling: *p* > 0.05 no marks; 0.01< *p* <0.05 labeled with an asterisk (*); 0.001< *p* <=0.01 labeled with two asterisks (**); *p* < 0.001 labeled with three asterisks (***).

The correlation between fermentation parameters, volatiles, and enzyme abundance in *Daqu* was analyzed based on Pearson (Figure 4). The fermenting ability was not correlated with the bacterial and fungal enzymes, while the saccharifying ability, liquefying ability, and esterifying ability was all affected by the enzymes. Liquefying ability and saccharifying ability affect the degradation and conversion of starch, there were more bacterial enzymes related to them, and the degree of coincidence was higher. Usually, *B. licheniformis* can secrete a large number of hydrolases such as amylase and acid protease [47], so it can notably increase the abundance of enzymes related to liquefying ability and saccharifying ability. The enzyme of fungal microbiota mainly had a high correlation with saccharifying ability, but it had a low correlation with liquefying ability and esterifying ability. There were only a few enzymes of the bacterial community related to alcohols, acids, aldehydes, ketones, alkanes and phenols, and most of them were related to the synthesis of esters and pyrazines. These results demonstrated that inoculating *B. licheniformis* significantly improves the synthesis of esters and pyrazines, and it regulated the ratio of the two metabolites. In FD, the abundance of ester-related enzymes decreased and that of pyrazine-related enzymes increased, mainly by increasing the three enzymes, including EC:1.1.1.100, EC:2.7.13.3, and EC:3.5.1.28, which averagely increased 22.50%, 21.85%, 182.37%, respectively. It was one of the main reasons for the increasing pyrazines content. There were 20 fungal enzymes related to metabolism, but they did not directly affect the synthesis of esters and pyrazines. The results of metabolic pathway analysis of related enzymes showed that the role of *B. licheniformis* was to increase the degradation and conversion of starch and fiber as well as to increase the rate of multiple pathways such as the TCA cycle, thus improving the production and accumulation of characteristic metabolites. *B. licheniformis* increased the expression abundance of EC:1.8.1.4 and EC:1.2.1.3 involved in multiple metabolic pathways in the bacterial community and EC:3.2.1.1 and EC:1.2.1.3 in the fungal community, in which EC:3.2.1.1 promoted starch degradation and EC:1.2.1.3 participated in multiple metabolic pathways. These changes made FD accumulate more volatiles than *Daqu* in the process.

## 4. Discussion

Inoculating functional microbes/consortia is one of the effective approaches to improving the product quality and characteristic flavor of traditional fermented food. It showed that metabolites in strong-flavor *Daqu*, especially esters and pyrazines (Figure 1), were improved when fortified by *B. licheniformis* J-49. In fact, the contribution of an isolate strain mainly included regulating the metabolic pathway of nitrogen metabolism notably (Figure 2 and Figure 4), resulting in increased content of NH4^+^ and promoting the synthesis of pyrazines (Figure 1). The abundance of histidine kinase (EC:2.7.13.3) expression as a signal transduction molecule [48] was increased, which might change the supply pattern of amino acids. This change significantly affected the synthetizing of aromatic active compounds during the process [49] even if one amino acid supplied was only shifted.

The correlation between the initial concentration of *B. licheniformis* inoculated and the fermentation parameters, as well as pyrazines, was non-linear, although the latter was also significantly enhanced (Figure 1). The contents of metabolites in sample D-6 were the highest in all FDs which related positively with the initial concentration, although the abundance of *Bacillus* identified was low (Figure 1a and Figure 2a). These results implied that the contribution of the initial concentration to the main physiochemical parameters as well as target metabolites might be related to the priority effect in the system [50]. The high concentration of inoculation made the target metabolites accumulate in a short time, but the by-products that accumulated at the same time may have a severe effect on community succession. The priority effect of functional microbes/consortia used for enhancement may be positive or negative due to different initial concentrations, which mainly depend on the interaction between the microbes and nutrient networks of the community [51]. In class H, *Bacillus* was negatively correlated with *Saccharopolyspora*, *Pseudonocardiaceae* and *Lactobacillus*. *Bacillus* was positively correlated with *Saccharopolyspora* and *Pseudonocardiaceae* in class M and L (Figure 3). The correlation between *Bacillus* and *Lactobacillus* was negative in class M and H but positive in class L. These results supported the above conjecture. *Saccharopolyspora* is one of the dominant actinomycetes in high-temperature *Daqu*, which is related to the fact that the high-temperature section and peak value of the matrix are slightly higher than those of medium and high-temperature *Daqu* (Figure 2). The antibacterial components secreted by *Saccharopolyspora* may inhibit *Bacillus* [52], so it was negatively correlated in class H (Figure 3).

In general, the bio-synthetizing of pyrazines is very complex, which the rate or content lies on the accumulating of intermediates such as 2,3-butanediol, acetoin, or L-threonine [53]. Therefore, it is an effective strategy to increase the content of pyrazines during the process whether a solid-state or liquid-state fermentation pattern develops a precursor supply strategy [54] or the engineered strains [55]. However, these strategies are difficult to apply for solid-state fermentation systems such as *Daqu* due to the possibility of process operation as well as biosafety. It showed that it was an effective way for biofortification based on inoculating the strains that produce high amounts of pyrazines [23,24,28]. It is worth noting that the results from *Daqu* were impacted by various factors, including strains specificity, interspecific interactions among the communities, interaction among nutrition networks, and micro-environmental condition. The activity of protease was enhanced by inoculating *B. licheniformis* and the content of L-threonine in the matrices. However, the propagation of *Bacillus* and synthesis of 3-hydroxy-2-butanone is more suitable under a weakly acidic environment [56], while the pyrazines synthesizing was suitable to proceed under a neutral environment.

In fact, the content of pyrazines in *Daqu* mainly depended on the strain specificity. As mentioned above, the content of pyrazines in FD was significantly enhanced when *B. licheniformis* or *B. subtilis* was isolated from high-temperature *Daqu*, while it was unobserved when the typical strain was inoculated [23,24]. Similar results were also obtained in our experiment. We screened two strains from the same source, *B. licheniformis* J-41 and *B. licheniformis* J-49 (Figure S1), respectively. The contents of key precursors, acetoin and 2,3-butanediol, were almost the same in the pure culture system composed of the sterilized wheat flour for both (Table S1). However, the significant discrepancy in the pyrazines content of *Daqu* that was manufactured in situ under identical process parameters was observed when the ranges of the initial concentration were from 2 × 10^5^ CFU/g to 8 × 10^6^ CFU/g (Figure 1 and Figure S2). The content of pyrazines in *Daqu* fortified with isolate J-49 was increased by 7.41, 2.35, 2.94, and 3.34 times than that with isolate J-41, respectively. In addition, pyrazines averagely increased 9.95 times by inoculation with *B. licheniformis* J-49 and 3.17 times by inoculation with *B. licheniformis* J-41 (Figure 1 and Figure S2). It was speculated that the strains specificity was related to the unsaturated fatty acids (palmitoleic acid 0.25% and oleic acid 0.19%) identified only in the cell membrane of isolate J-49 (Table S2), and it improved their fluidity of response to the stress [57]. The abundance of *Bacillus* had not increased in *Daqu* by *B. licheniformis* J-41 (A-1–A-5), which was similar to sample B (Figure 2). Meanwhile, the abundance of acid-producing bacteria was significantly induced with the initial inoculation concentration, and the amplitude reduction by isolate J-49 was higher than that by isolate J-41 (Figure S3). These results suggested that the synthesis rate of pyrazines was also closely related to the acidification rate in the matrices, especially in the pre-stage [58], and the latter lay on the interspecific interactions of isolated from other species and genera. When the initial concentration was up to 2 × 10^7^ CFU/g, the content of pyrazines was decreased in *Daqu* with isolate J-49 (Figure 1). The high inoculation concentration significantly increased hydrolase activity, improved the degradation of starch and protein, and enhanced the propagation rate of the community and synthesis rate of target products in the pre-stage. Meanwhile, the by-products that formed largely weakened the inhibition of *Bacillus* to acid-producing bacteria, resulting in decreased pyrazines content and fermenting ability. It might increase the negative correlation of species and genera among communities or core modules (Figure 3) due to the excessive disturbing by isolate J-49 [59]. In contrast, it may be the inhibition of acid-producing bacteria for the latter which needs to explore further.

The dominant fungi in sample B were *Pichia* and *Rhizomucor* (Figure 2b). Inoculating *B. licheniformis* to enhance the fermentation of the *Daqu* matrix may increase the temperature quickly in the initial stage, which resulted in increasing the abundance of *Thermomyces* and *Thermoascus* in FDs (Figure 2b). The growth and reproduction rate of the main communities increased along with the accumulation of bio-heat and significantly changed the composition of fungal communities [15]. *Thermoascus* and *Thermomyces* secreted high-temperature hydrolase, such as xylanase and cutinase, which increased the rate of substrate hydrolysis and transformation. Meanwhile, its metabolites fed back the metabolism of *B. licheniformis* to increase the synthetic content of characteristic flavor components. The thermostable hydrolase secreted by *Thermoascus* not only improves the hydrolysis rate of the substrate in a high-temperature environment [60] but also benefits the rate of non-enzymatic reactions such as Maillard to synthesize pyrazines. In FD, the abundance of hemicellulose hydrolase secreted by *Thermomyces* [61], especially EC:3.2.1.1, was significantly increased (Figure 4), and the degradation of starch, chitin, and xylan was also notably increased, which provided the necessary intermediates for the synthesis of pyrazines (Figure 1 and Figure 2).

*S. cerevisiae* can inhibit the growth of *B. licheniformis* but not vice versa [62]. Increasing the initial concentration of *B. licheniformis* in *Qupei* is beneficial to increase the content of volatiles such as aromatic compounds, volatile acids, pyrazine, etc. (Figure 1) [21]. Fermenting ability is characterized by the increment of CO_2_ during the initial phase, which is closely related to the community composition in *Daqu*. Except for sample D-6, one of the contributions of *B. licheniformis* was to improve the fermenting ability. Although the former had the highest liquefying ability and saccharifying ability, its fermenting ability was significantly lower than that of other *Daqu* (Table 1). It may be related to the high ratio of liquefying ability to saccharifying ability, and the strong gelatinization ability and cellulose degradation ability of the starch is due to the high liquefying ability. The strong hydrolysate ability to be converted into small molecular fermentable sugar leads to the high saccharifying ability. The former was high and the latter was low, which caused an excessive accumulation of dextrin and small molecular cellulose hydrolysates, increased the viscosity of the matrix, affected mass transfer and dissolved oxygen concentration, and slowed down the reproduction of aerobic consortia. It was suitable for the growth and metabolism of facultative anaerobic consortia, such as lactic acid bacteria, and the accumulation of acidic components such as organic acids. This inhibited the fermentation rate of monosaccharide conversion to ethanol by yeast and reduced the fermenting ability. The result of the acidity in sample D-6 supported this speculation (Table 1, Figure 4).

In this study, the strains that originated from *Daqu* powder mutated in space not only significantly increased the content of its characteristic flavor, such as pyrazine and 2,3-butanediol, but also shifted the microbial community structure in FD and improved their fermentation parameters. Among them, the initial concentration and strains specificity are the important factors on the *Daqu* community and quality.

## Figures and Tables

**Table 1:** Difference of physicochemical properties among samples.

Samples	Inoculum Concentrations (CFU/g)	Moisture (%)	Acidity (mmol/10 g)	Liquefying Ability (g/g·h)	Saccharifying Ability (mg/g·h)	Fermenting Ability (g/0.5 g·72 h)	Esterifying Ability (mg/50 g·7 d)
B	0	10.16 ± 0.00 ^a^	0.66 ± 0.01 ^c^	0.92 ± 0.03 ^bc^	505.27 ± 13.93 ^f^	0.17 ± 0.01 ^c^	5.35 ± 0.69 ^a^
D-1	8 × 10^4^	9.47 ± 0.00 ^a^	0.75 ± 0.02 ^bc^	1.64 ± 0.27 ^a^	935.99 ± 9.03 ^b^	0.21 ± 0.00 ^b^	1.31 ± 0.07 ^d^
D-2	2 × 10^5^	9.59 ± 0.00 ^a^	0.92 ± 0.02 ^b^	0.75 ± 0.04 ^c^	748.98 ± 15.70 ^c^	0.23 ± 0.00 ^b^	2.16 ± 0.48 ^cd^
D-3	8 × 10^5^	9.71 ± 0.01 ^a^	0.70 ± 0.00 ^c^	0.91 ± 0.05 ^bc^	654.87 ± 6.06 ^e^	0.30 ± 0.01 ^a^	2.35 ± 0.05 ^c^
D-4	2 × 10^6^	9.84 ± 0.01 ^a^	0.72 ± 0.01 ^c^	1.05 ± 0.11 ^bc^	706.94 ± 1.45 ^d^	0.20 ± 0.02 ^bc^	4.08 ± 0.92 ^b^
D-5	8 × 10^6^	9.69 ± 0.00 ^a^	0.78 ± 0.02 ^bc^	1.21 ± 0.12 ^b^	736.30 ± 3.14 ^c^	0.21 ± 0.03 ^bc^	4.69 ± 0.10 ^ab^
D-6	2 × 10^7^	9.84 ± 0.00 ^a^	1.10 ± 0.02 ^a^	1.83 ± 0.12 ^a^	985.71 ± 15.99 ^a^	0.10 ± 0.02 ^d^	4.17 ± 0.11 ^b^

Note: B, the unfortified of *B. licheniformis* in *Daqu*. D-1–D-6: the inoculation of *Daqu*. Different letters in the same column of a–f represent significant differences (*p* < 0.05).

**Table 2:** Difference of microbial community α-diversity indexes among *Daqu* samples.

No.	Abundance Indexes				Diversity Indexes			
	Chao1		Observed Species		Shannon		Simpson	
	Bacteria	Fungi	Bacteria	Fungi	Bacteria	Fungi	Bacteria	Fungi
B	712.40	72.14	670.50	71.80	4.52	2.99	0.86	0.84
D-1	402.36	47.44	322.40	46.50	4.14	0.45	0.85	0.10
D-2	405.25	93.30	346.60	92.60	3.16	2.33	0.77	0.65
D-3	478.27	55.99	384.60	55.90	3.96	1.51	0.85	0.46
D-4	471.98	49.30	361.00	49.00	3.99	1.14	0.88	0.32
D-5	951.45	55.16	842.70	54.80	5.91	1.41	0.95	0.39
D-6	936.66	46.05	867.80	45.90	5.76	0.92	0.92	0.23

## Data Availability

Data are contained within the article or supplementary material.

## Supplementary Materials

The following supporting information can be downloaded at: https://www.mdpi.com/article/10.3390/foods12020304/s1, Table S1: Concentration of 2,3-butanediol (mg/kg) in strains of *B. licheniformis* J-41 and *B. licheniformis* J-49; Table S2: Fatty acid content in cell membrane of *B. licheniformis* J-41 and *B. licheniformis* J-49; Figure S1: Microscopic graphs of colonies and gram staining of *B. licheniformis* J-41 and *B. licheniformis* J-49; Figure S2: Metabolites of fortified *Daqu* using *B. licheniformis* J-41 (A-1–A-6). *Daqu* (a) bacterial and (b) fungal community composition. Volatile metabolites (c) content and (d) abundance of *Daqu*; Figure S3: Profiles of *Bacillus* and acid-producing bacteria in *Daqu*.
