# Supplementary material for: Mechanism of Enhancing Pyrazines in Daqu via Inoculating Bacillus licheniformis with Strains Specificity
Source: Foods. 2023 Jan 9;12(2):304. doi: 10.3390/foods12020304 (PMC9858619; doi:10.3390/foods12020304)
Supplement: Supplementary file 1 [file foods-12-00304-s001.zip › foods-2093223-supplementary.pdf]

foods

Supplementary Materials for the Paper (Article)

Mechanism of Enhancing Pyrazines in *Daqu* via Inoculating *Bacillus licheniformis* with Strains Specificity

Qiuxiang Tang [1], Xiaoru Chen [1], Jun Huang [1], Suyi Zhang [2], Hui Qin [2], Yi Dong [2], Chao Wang [2], Xiaojun Wang [2], Chongde Wu [1], Yao Jin[1], Rongqing Zhou [1,3,*]

[1] College of Biomass Science and Engineering, Sichuan University, Chengdu 610065, China; Qiuxiang Tang,
[2] Luzhou Laojiao Company Limited, Luzhou 646000, China; Suyi Zhang,
[3] National Engineering Research Centre of Solid-state Brewing, Luzhou 646000, China; Rongqing Zhou,
* Correspondence: Rongqing Zhou, zhourqing@scu.edu.cn; Tel. +86-28-85406149: (College of Biomass Science and Engineering, Sichuan University, Chengdu 610065, China)

Table S1. Concentration of 2, 3-butanediol (mg/kg) in strains of B. licheniformis J-41 and B. licheniformis J-49.

Generations	J-41	J-49
2	172.32 ± 10.12	176.31 ± 14.27
3	74.95 ± 3.40	143.88 ± 5.44
4	105.31 ± 2.13	164.61 ± 7.82
5	114.93 ± 27.48	246.93 ± 4.82
6	100.53 ± 5.23	168.37 ± 12.24
7	88.16 ± 8.55	112.28 ± 7.80
8	89.64 ± 6.55	120.51 ± 1.73

Table S2. Fatty acid content in cell membrane of *B. licheniformis* J-41 and *B. licheniformis* J-49.

Fatty acid (%)	J-41	J-49
i-C13:0	0.48 ± 0.10	0.40 ± 0.26
i-C14:0	38.87 ± 0.06	33.37 ± 0.16
anteiso-C14:0	25.16 ± 0.18	28.02 ± 0.12
C16:0	6.03 ± 0.05	6.25 ± 0.06
anteiso-C16:0	12.59 ± 0.09	15.02 ± 0.08
C16:1	#NA	0.25 ± 0.05
C17:0	15.86 ± 0.05	16.15 ± 0.10
i-C17:0	1.02 ± 0.01	0.79 ± 0.01
C18:1ω9	#NA	0.19 ± 0.01

Note: #NA means that it is not detected.

Citation: Tang, Q.; Chen, X.; Huang, J.; Zhang, S.; Qin, H.; Dong, Y.; Wang, C.; Wang, X.; Wu, C.; Jin, Y.; et al. Mechanism of Enhancing Pyrazines in *Daqu* via Inoculating *Bacillus licheniformis* with Strains Specificity. *Foods* **2023**, *12*, 304. https://doi.org/10.3390/foods12020304

Academic Editors: Hasan Tanguler, Bulent Kabak

Received: 28 November 2022
Revised: 3 January 2023
Accepted: 4 January 2023
Published: 9 January 2023

Copyright: © 2023 by the authors. Licensee MDPI, Basel, Switzerland. This article is an open access article distributed under the terms and conditions of the Creative Commons Attribution (CC BY) license (https://creativecommons.org/licenses/by/4.0/).

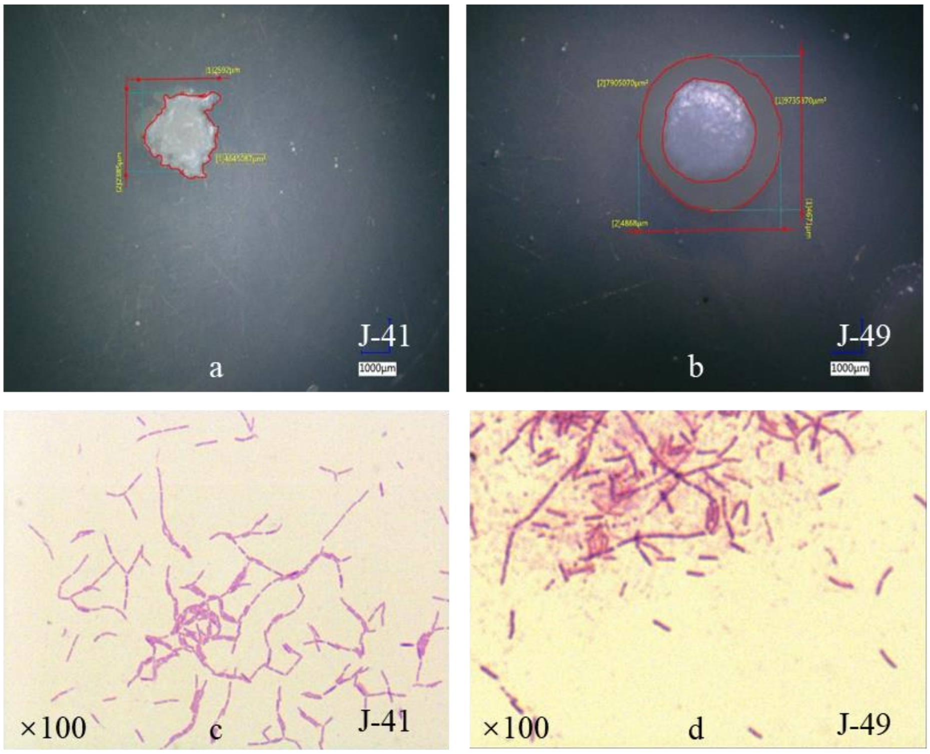

Figure S1. Microscopic graphs of colonies and gram staining of *B. licheniformis* J-41 and *B. licheniformis* J-49.

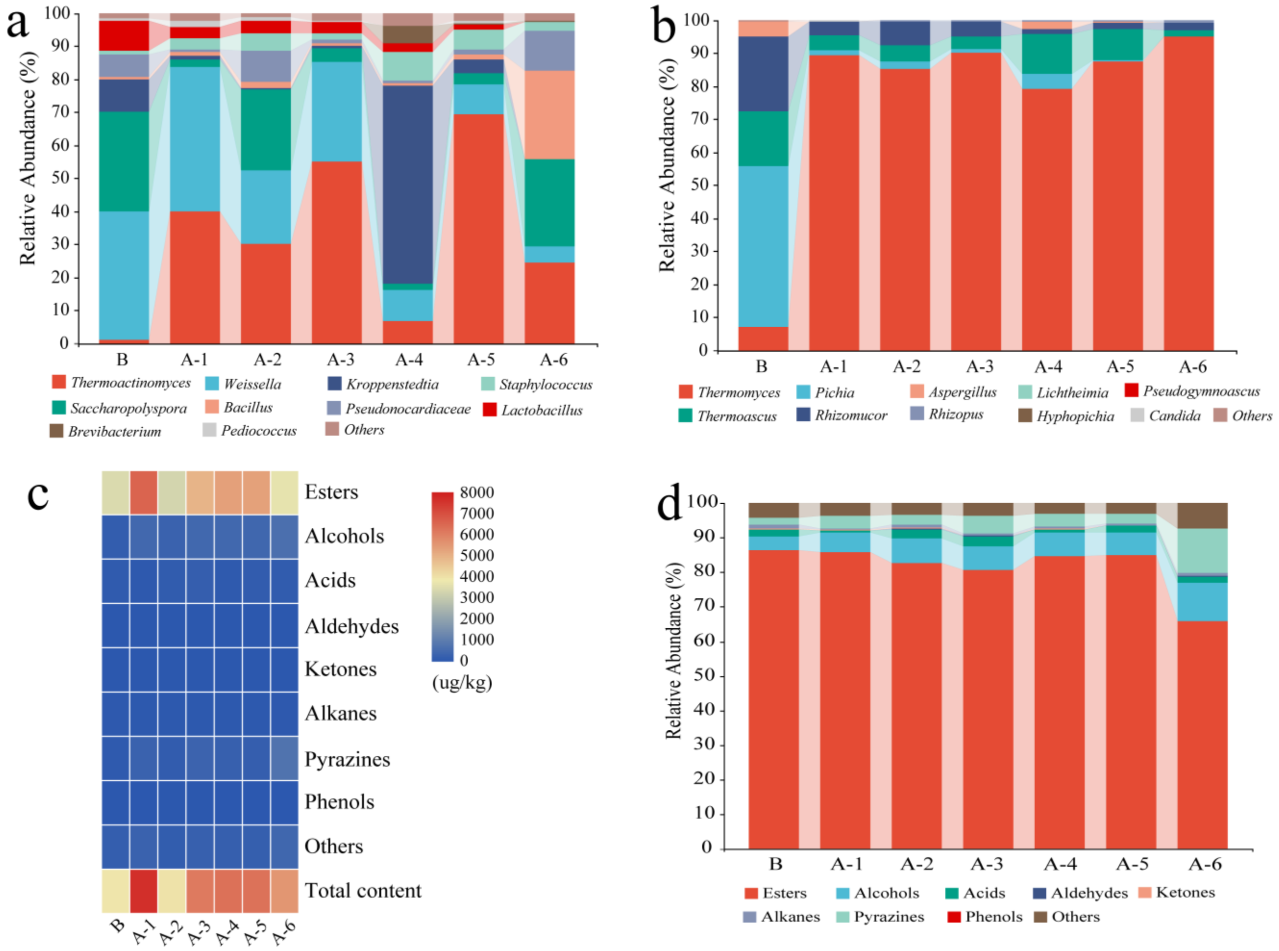

Figure S2. Metabolites of fortified *Daqu* using *B. licheniformis* J-41 (A-1–A-6). *Daqu* (a) bacterial and (b) fungal community composition. Volatile metabolites (c) content and (d) abundance of *Daqu*.

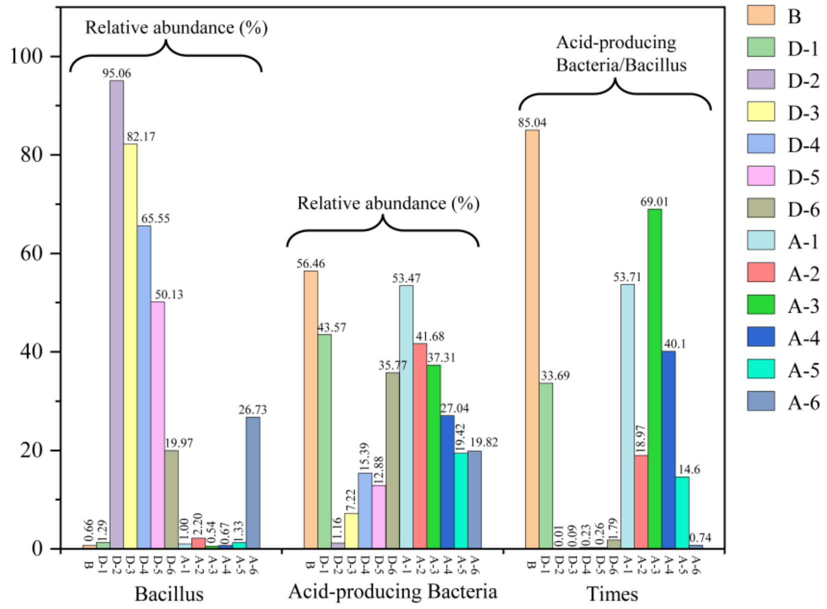

Figure S3. Profiles of *Bacillus* and acid-producing bacteria in *Daqu*.
